# Cognitive ability and risk of death from lower respiratory tract infection: findings from UK Biobank

**DOI:** 10.1038/s41598-018-38126-w

**Published:** 2019-02-04

**Authors:** Catharine R. Gale, Ian J. Deary, G. David Batty

**Affiliations:** 1 MRC Lifecourse Epidemiology Unit, University of Southampton, Southampton, UK; 2 Centre for Cognitive Ageing and Cognitive Epidemiology, Department of Psychology, University of Edinburgh, Edinburgh, UK; 3 Department of Psychology, University of Edinburgh, Edinburgh, UK; 4 Department of Epidemiology and Public Health, University College, London, UK

## Abstract

Dementia increases the risk of lower respiratory tract infection, but it is unclear whether risk varies across the normal range of cognitive ability. People with higher cognitive ability tend to behave in a healthier fashion as regards risk factors for lower respiratory tract infection and there is evidence that they have a lower risk of dying from respiratory disease as a whole. We therefore investigated the relationship between cognitive ability and mortality from lower respiratory tract infection. Participants were 434,413 people from UK Biobank (54% female). Cognitive ability was measured using tests of reaction time and reasoning. Data on deaths from lower respiratory infection were obtained from death certificates. Over a mean follow-up period of 6.99 years, 1,282 people died of lower respiratory infection. Mortality from lower respiratory tract infection fell as cognitive ability increased. For a standard deviation faster reaction time, the age- and sex-adjusted hazard ratio (95% confidence interval) was 0.80 (0.76, 0.83) and the multivariable-adjusted hazard ratio was 0.87 (0.83, 0.91). There were similar though weaker associations when cognitive ability was assessed using a reasoning test. These findings suggest that variation across the normal range of cognitive ability increase risk of dying from lower respiratory tract infection.

## Introduction

Estimates from the Global Burden of Disease Study indicate that, in 2015, lower respiratory tract infections were the leading infectious cause of death and the fifth-leading cause of death overall, responsible for 2.74 million deaths^1^. Lower respiratory tract infection is largely preventable. Smoking, poor oral or hand hygiene, chronic conditions such as cardiovascular disease, diabetes, chronic obstructive pulmonary disease, and dementia are associated with an increased risk^2–4^.

It is unclear whether variation in cognitive abilities across the normal range is linked with risk of lower respiratory tract infection, but such a link seems plausible. Cognitive abilities are those mental functions that involve the storage, selection, manipulation and organisation of information. They can be assessed using standardised tests of specific cognitive abilities such as reasoning, processing speed, memory, or visuospatial ability. People vary in how quickly and accurately they can carry out such mental tasks. One of the most replicated findings in psychology is that scores on tests of cognitive abilities tend to be positively correlated, such that people who perform well on a test of one cognitive ability tend to perform well on tests of other cognitive abilities. This is led to the use of the term ‘general cognitive ability’ or ‘general intelligence’ (usually known as *g*). Some individual cognitive tests provide a good measure of general cognitive ability, but usually it is assessed using a battery of tests requiring different types of cognitive ability^5^. There is now a large body of evidence that individuals with higher general cognitive ability in childhood or early adulthood have a lower risk of dying from all causes, cardiovascular disease, and some cancers^6–8^. Studies of all-cause mortality show that the association between higher ability and lower risk of death is graded across the full range of cognitive ability^9^. People with higher cognitive ability are better equipped to obtain, process, and respond to health promotion information^10^. They tend to behave in a healthier fashion as regards smoking and oral hygiene^11,12^. Higher educational attainment—a strong correlate of cognitive ability—is consistently associated with take-up of influenza and pneumococcal vaccination^13^. Whether cognitive ability influences prognosis after onset of lower respiratory tract infection is unknown, but the finding that a standard deviation advantage in cognitive ability in childhood was associated with a 28% reduction in risk of death from respiratory disease overall during 68 years of follow-up^7^ suggests that it is likely to be linked to reduced mortality from lower respiratory tract infection.

The UK Biobank is a very large general population prospective study, set up as a resource to investigate genetic and non-genetic risk factors for major diseases in middle and old age^14^. Participants underwent a detailed phenotypic assessment at baseline and their health is being monitored, primarily through linkage to routinely collected national data. We used data from UK Biobank to investigate the relationship between cognitive ability—as indicated by reaction time (a measure of processing speed) and reasoning—at baseline and risk of death from lower respiratory tract infection, taking account of potential confounding factors, namely age, sex, psychological distress, smoking status, diagnoses of various chronic diseases, number of medications, and socioeconomic status, as measured by educational attainment and Townsend deprivation index.

## Methods

### Participants

Between 2006 and 2010, 502,655 community-dwelling people aged between 37 and 73 years and living in the United Kingdom were recruited to UK Biobank and took part in the baseline survey^15^. They completed questionnaires to provide information on demographics, lifestyle, and medical history, underwent cognitive and physical assessments, and agreed to have their health followed longitudinally. Approval for UK Biobank was received from the North-West Multi-centre Research Ethics Committee (REC reference 11/NW/0382). The research was carried out in accordance with the Declaration of Helsinki of the World Medical Association and participants gave written informed consent.

### Measures

#### Cognitive ability

Reaction time was measured using a computerized Go/No-Go “Snap” game. Participants were presented with two cards with symbols on them. If the cards were identical, participants were instructed to push the button-box as quickly as possible with their dominant hand. Twelve pairs of cards were shown. The first five pairs were used as a practice. Of the remaining seven pairs, four contained identical cards. Reaction time score was the mean time in milliseconds to press the button when one of these four pairs was presented. Internal consistency of the four test trials was high (Cronbach’s α = 0.85). Choice reaction time means have a strong loading on a latent trait of general mental ability (*g*)^16^ and they also correlate strongly with single mental tests that involve complex reasoning and knowledge and which are strongly *g*-loaded^17^. Therefore, as is the case for any other cognitive test, reaction time’s associations with health outcomes could be due to *g*–for which they can be a proxy–or to some specific aspect of reaction time variance.

Verbal and numerical reasoning was measured using a computerized 13-item multiple-choice test with a two-minute time limit. The score was the number of correct answers. This test was introduced after the start of the baseline assessment so data are available for a subset only.

#### Mortality ascertainment

Death certificate data were available for deaths occurring up to 22^nd^ February 2016. We examined mortality from lower respiratory tract infection identified by International Classification of Disease 10th revision codes J09-18 (influenza and pneumonia) and J20-22 (other acute lower respiratory infections). Any mention of these on the certificate was counted as death from lower respiratory tract infection.

### Covariates

In addition to age and sex, we chose smoking status, diagnoses of chronic disease, psychological distress, number of medications taken, highest educational qualification, and Townsend Deprivation Index at the baseline assessment as covariates. During interview participants provided information about smoking status (never, ex- or current smoker), highest educational qualification (categorized in seven groups, ranging from no qualifications to degree), number of medications taken, and whether they had been diagnosed by a physician with vascular or heart problems, diabetes, cancer, chronic bronchitis or emphysema, or asthma. Participants completed the 4-item version of the Patient Health Questionnaire to assess psychological distress^18^. Items are rated on a 4-point Lickert scale, such that total potential scores range from 0 to 12, with higher scores indicating greater psychological distress. The Townsend deprivation index is an area-based measure of deprivation based on levels of unemployment, non-car ownership, non-home ownership, and household overcrowding. It was calculated immediately before participants joined UK Biobank, using data from the preceding national census. Participants were assigned a score corresponding to the postcode of their home. Negative values indicate higher socioeconomic status.

### Statistical analysis

We used t-tests or Chi-square tests to examine the characteristics of the participants according to whether they died from lower respiratory tract infection during follow-up. Cox proportional hazards regressions were used to examine mortality from lower respiratory tract infection. Survival time in days was calculated from date of attendance at the Assessment Centre to date of death or 22nd February 2016, whichever occurred first. We calculated unadjusted hazard ratios (HRs) (95% confidence intervals) (CIs) to show the relationship between all baseline characteristics and risk of death from lower respiratory tract infection during follow-up. For the multivariable Cox models, we calculated hazard ratios for a standard deviation decrease in reaction time or a standard deviation increase in reasoning, adjusted first for age and sex, and then with further adjustment for smoking status, psychological distress (as a continuous variable), number of medications (as a continuous variable), diagnosis of vascular or heart problems, diabetes, cancer, chronic bronchitis or emphysema, or asthma, educational attainment and Townsend deprivation index.

### Analytical sample

Of the 502,655 people recruited to UK Biobank, 434,413 had complete data on reaction time and the covariates (234,609 women and 199,804 men). A subset of 146,513 had complete data on reasoning and the covariates.

## Results

In total, 1,282 of the 434,413 participants died of lower respiratory tract infection during a mean follow-up of 6.99 years. Table 1 describes the baseline characteristics of the study participants according to whether they died from lower respiratory tract infection. In this large sample all characteristics were significantly associated with mortality risk at p < 0.0001, such that death from lower respiratory tract infection was associated with older age, being male, smoking, lack of educational qualifications, greater deprivation, having a diagnosis of vascular or heart problems, diabetes, chronic bronchitis or emphysema, asthma or cancer, high levels of psychological distress, taking 5 or more medications, and poorer cognitive ability as indicated by slower reaction time and lower scores for reasoning.

**Table 1:** Baseline characteristics of the participants according to death from lower respiratory tract infection (n = 434,413).

Characteristic^a^	Died of lower respiratory infection		
	Yes (n = 1,282)	No (n = 433,131)	Unadjusted hazard ratio^b^ (95% CI)
Age (yrs), mean (SD)	62.3 (6.05)	56.5 (8.07)	2.58 (2.39, 2.78)
Male, no. (%)	850 (66.3)	199,954 (45.9)	2.33 (2.08, 2.62)
Reaction time, mean (SD)	609.9 (143.8)	556.8 (115.5)	0.73 (0.70, 0.75)
Reasoning, mean (SD)^c^	5.51 (2.05)	6.13 (2.13)	0.75 (0.67, 0.84)
Current smoker, no. (%)	297 (23.2)	43,935 (10.4)	2.68 (2.35, 3.05)
Vascular or heart disease, no. (%)	656 (51.2)	126,034 (29.1)	2.58 (2.31, 2.87)
Diabetes, no. (%)	171 (13.3)	21,514 (4.97)	3.05 (2.58, 3.57)
Chronic bronchitis or emphysema, no. (%)	140 (10.9)	6,682 (1.54)	7.97 (6.69, 9.50)
Asthma, no. (%)	197 (15.4)	49,859 (11.5)	1.39 (1.20, 1.62)
Cancer, no. (%)	259 (20.2)	33,252 (7.68)	3.17 (2.77, 3.63)
On ≥ 5 medications, no. (%)	603 (47.0)	75,986 (17.5)	4.14 (3.72, 4.62)
Psychological distress score ≥ 3, no. (%)	409 (31.9)	102,476 (23.7)	1.60 (1.40, 1.83)
No educational qualifications, no. (%)	461 (36.0)	67,981 (15.7)	2.96 (2.67, 3.28)
Townsend score, mean (SD)	−0.48 (3.42)	−1.42 (3.02)	1.34 (1.27, 1.41)

^a^All differences in characteristics between those who did or did not die of lower respiratory infection were significant at p < 0.0001. ^b^Hazard ratios for the continuous variables are expressed for a standard deviation (SD) increase in the variable, with the exception of reaction time which is for a SD decrease so it is consistent with the analyses shown in Table 2. ^c^Data on reasoning is based on a subset of 146,513 participants of whom 331 died of lower respiratory tract infection.

Table 2 shows HRs (95% CIs) for risk of death from lower respiratory tract infection according to reaction time. Results are presented according to thirds of the distribution of reaction time and for a standard deviation (SD) reduction in reaction time. In age- and sex-adjusted analysis, risk fell with faster reaction time: compared to those whose reaction time was in the slowest third of the distribution, participants whose reaction time was in the quickest third of the distribution had a HR (95% CI) of 0.57 (0.49, 0.66). Further adjustments in turn for smoking, psychological distress, diagnoses of chronic diseases and number of medications, and the measures of socioeconomic status (highest educational qualification and Townsend deprivation index) slightly attenuated this association, but it remained statistically significant. After adjustment for all covariates, compared to those whose reaction time was in the slowest third of the distribution, participants whose reaction time was in the quickest third of the distribution had a HR (95% CI) of 0.70 (0.60, 0.81). For a SD faster reaction time, the age- and sex-adjusted HR (95% CI) was 0.80 (0.76, 0.83). The fully-adjusted HR for a SD faster reaction time was 0.87 (0.83, 0.91).

**Table 2:** Hazard ratios (95% CI) for Mortality from Lower Respiratory Tract Infection according to Reaction Time in UK Biobank Participants (n = 434,413).

Adjustments	Reaction time categories						Per SD reduction	
	1 (Quickest) n = 144,362 (254 deaths)		2 n = 143,582 (363 deaths)		3 (Slowest) n = 145,187 (665 deaths)	*P* for linear trend		
	HR	95% CI	HR	95% CI	HR		HR	95% CI
Age & sex	0.57	0.49, 0.66	0.64	0.57, 0.74	1.0	<0.0001	0.80	0.76, 0.83
Age, sex, and smoking status	0.60	0.51, 0.69	0.66	0.58, 0.75	1.0	<0.0001	0.81	0.77, 0.84
Age, sex, smoking status and psychological distress	0.63	0.54, 0.73	0.68	0.60, 0.78	1.0	<0.0001	0.83	0.79, 0.86
Age, sex, smoking status, psychological distress, diagnoses of chronic disease & number of medications	0.67	0.57, 0.77	0.71	0.63, 0.81	1.0	<0.0001	0.85	0.82, 0.89
Age, sex, smoking status, diagnoses of chronic disease, number of medications, educational attainment & Townsend deprivation index	0.70	0.60, 0.81	0.74	0.64, 0.84	1.0	<0.0001	0.87	0.83, 0.91

In the subset of 146,513 participants with data on reasoning, there were 331 deaths from lower respiratory tract infection. Table 3 shows HRs (95% CIs) for risk of death from lower respiratory tract infection. Results are presented according to thirds of the distribution of reasoning score and for a standard deviation (SD) increase in it. In age- and sex-adjusted analysis, risk fell as score for reasoning increased: compared to those whose score in the lowest third of the distribution, people with a score in the highest third of the distribution had a HR (95% CI) of 0.66 (0.51, 0.87). This association was weakened by further adjustment for smoking and psychological distress, became non-significant after adjustment for diagnoses of chronic diseases and number of medications, and was further attenuated after additional adjustment for the two measures of socioeconomic status, highest educational qualification and Townsend deprivation index: HR 1.07 (0.80, 1.44). For a SD increment in reasoning, the age- and sex-adjusted and fully-adjusted HRs were 0.78 (0.70, 0.88) and 0.96 (0.85, 1.09), respectively.

**Table 3:** Hazard ratios (95% CI) for Mortality from lower respiratory tract infection according to Verbal-Numerical Reasoning Test Score in UK Biobank Participants (n = 146,513).

Adjustments	Reasoning categories						Per SD reduction	
	1 (Highest) n = 62,141 (109 deaths)		2 n = 49,069 (120 deaths)		3 (Lowest) n = 34,972 (102 deaths)	*P* for linear trend		
	HR	95% CI	HR	95% CI	HR		HR	95% CI
Age & sex	0.66	0.50, 0.86	0.88	0.68, 1.14	1.0	0.002	0.78	0.70, 0.88
Age, sex, and smoking status	0.69	0.53, 0.91	0.90	0.69, 1.17	1.0	0.007	0.80	0.72, 0.90
Age, sex, smoking status and psychological distress	0.75	0.57, 0.99	0.96	0.74, 1.25	1.0	0.036	0.83	0.74, 0.93
Age, sex, smoking status, psychological distress, diagnoses of chronic disease & number of medications	0.86	0.65, 1.12	1.05	0.80, 1.37	1.0	0.251	0.88	0.79, 0.98
Age, sex, smoking status, diagnoses of chronic disease, number of medications, educational attainment & Townsend deprivation index	1.07	0.80, 1.44	1.17	0.89, 1.53	1.0	0.638	0.96	0.85, 1.09

## Discussion

We found that people with higher cognitive ability, as indicated by faster reaction time, had a markedly reduced risk of dying from lower respiratory tract infection. Similar associations were found when cognitive ability was assessed using reasoning scores, though here the relationship was weaker and was attenuated by adjustment for health at baseline, as indicated by diagnoses of chronic diseases and number of medications, and in particular by adjustment for measures of socioeconomic status, namely highest educational qualification and Townsend deprivation index. This might be an over-adjustment as educational attainment could reflect cognitive ability^19^.

Smoking explained little of the relationship between either measure of cognitive ability and risk of death from lower respiratory tract infection. Poorer health, as measured by diagnoses of various chronic diseases and number of medications, accounted for a small part of the association between reaction time and mortality from lower respiratory tract infection, but had a stronger attenuating effect on the association between reasoning and mortality from lower respiratory tract infection.

Oral or hand hygiene might play some part in the association. People with higher cognitive ability are more likely to behave in a healthier fashion, and that includes oral hygiene^11,12^. Individuals who are more highly educated—a strong correlate of cognitive ability—have a higher take-up of influenza and pneumococcal vaccination^13^. We were unable to explore the potential role of uptake of the influenza or pneumococcal vaccines in these data.

Strengths of our study include the large sample size and number of deaths. The study also has some limitations. The first of these relates to the tests of cognitive ability. Reasoning was measured on a subset of participants, using a very short, bespoke test and mean reaction time was based on only four trials. The brevity of the cognitive tests was due to the need to conduct computerized assessments in very large numbers of participants. Using two or more tests to assess each cognitive ability is likely to have provided more accurate measures, although it is worth noting that scores on the UK Biobank tests of reasoning and reaction time have been shown to be predictive of all-cause mortality^20^. That finding, coupled with the observations of the current study, confirms that these cognitive tests provide valid measures despite their brevity. The second limitation of our study relates to lack of information on some factors that might potentially confound the relationship between cognitive ability at baseline and mortality from lower respiratory tract infection. We had no data on whether participants had been vaccinated against pneumococcal infections or whether they had an annual influenza vaccination, and information on whether they might be immunosuppressed was limited. We were able to adjust for a diagnosis of cancer, along with diagnoses of various other chronic diseases, but there was no data at baseline on diagnoses of HIV or AIDS. We were unable to take account specifically of sleeping pill use, though we did adjust for total number of medications used. Thirdly, there is evidence of a ‘healthy volunteer’ selection bias in UK Biobank, as in other volunteer-based cohort studies^21^. However, this lack of representativeness of the general population is not a limitation when it comes to investigating risk factor/disease associations and publications using the data have shown expected associations with mortality^22^. Finally, it is important to consider whether our results might have arisen due to reverse causation, whereby the presence of lower respiratory infection at the time of the baseline assessment might have affected performance on the cognitive tests. This seems unlikely to explain our findings. We had no information on the presence of acute respiratory infection during the baseline assessment, but we took account of diagnoses of chronic bronchitis or emphysema at baseline and our outcome was death from influenza, pneumonia or other acute lower respiratory infections.

In this large study of older adults, we found some evidence that variation across the normal range of cognitive ability is associated with risk of dying from lower respiratory tract infection. Identifying the underlying mechanisms may add to knowledge of the aetiology of this largely preventable condition.

## Data Availability

The data used in this study are owned by UK Biobank. Applications to use the data should be made to UK Biobank (www.ukbiobank.ac.uk).

## References

[1] GBD 2015 LRI Collaborators. Estimates of the global, regional, and national morbidity, mortality, and aetiologies of lower respiratory tract infections in 195 countries: a systematic analysis for the Global Burden of Disease Study 2015. *Lancet Infect Dis* **17**, 1133–1161 (2017). 10.1016/S1473-3099(17)30396-1 PMC5666185 28843578

[2] Torres A, Peetermans WE, Viegi G, Blasi F (2013). Risk factors for community-acquired pneumonia in adults in Europe: a literature review. Thorax.

[3] Vinogradova Y, Hippisley-Cox J, Coupland C (2009). Identification of new risk factors for pneumonia: population-based case-control study. The British journal of general practice: the journal of the Royal College of General Practitioners.

[4] Almirall J (2008). New evidence of risk factors for community-acquired pneumonia: a population-based study. Eur Respir J.

[5] Deary, I. J. *Looking down on human intelligence: from psychometrics to the brain*. Oxford University Press (2000).

[6] Calvin CM (2011). Intelligence in youth and all-cause-mortality: systematic review with meta-analysis. Int J Epidemiol.

[7] Calvin CM (2017). Childhood intelligence in relation to major causes of death in 68 year follow-up: prospective population study. BMJ.

[8] Christensen GT, Mortensen EL, Christensen K, Osler M (2015). Iq in Youth and Later Life All-Cause and Cause-Specific Mortality in the Danish Conscription Database. Gerontologist.

[9] Cukic I, Brett CE, Calvin CM, Batty GD, Deary IJ (2017). Childhood IQ and survival to 79: Follow-up of 94% of the Scottish Mental Survey 1947. Intelligence.

[10] Gottfredson LS (2004). Intelligence: Is it the epidemiologists’ elusive “Fundamental cause” of social class inequalities in health?. Journal of Personality and Social Psychology.

[11] Wraw C, Der G, Gale CR, Deary IJ (2018). Intelligence in youth and health behaviours in middle age. Intelligence.

[12] Sabbah W, Sheiham A (2010). The relationships between cognitive ability and dental status in a national sample of USA adults. Intelligence.

[13] La EM (2018). An analysis of factors associated with influenza, pneumoccocal, Tdap, and herpes zoster vaccine uptake in the US adult population and corresponding inter-state variability. Hum Vaccin Immunother.

[14] Collins R (2012). What makes UK Biobank special?. Lancet.

[15] Sudlow C (2015). UK biobank: an open access resource for identifying the causes of a wide range of complex diseases of middle and old age. PLoS Medicine.

[16] Johnson W, Deary IJ (2011). Placing inspection time, reaction time, and perceptual speed in the broader context of cognitive ability: The VPR model in the Lothian Birth Cohort 1936. Intelligence.

[17] Deary IJ, Der G, Ford G (2001). Reaction times and intelligence differences - A population-based cohort study. Intelligence.

[18] Kroenke K, Spitzer RL, Williams JB, Lowe B (2009). An ultra-brief screening scale for anxiety and depression: the PHQ-4. Psychosomatics.

[19] Deary IJ, Strand S, Smith P, Fernandes C (2007). Intelligence and educational achievement. Intelligence.

[20] Yates T (2018). Reaction time, cardiorespiratory fitness and mortality in UK Biobank: An observational study. Intelligence.

[21] Fry A (2017). Comparison of sociodemographic and health-related characteristics of UK Biobank participants with those of the general population. American Journal of Epidemiology.

[22] Ganna, A. & Ingellson, E. 5 year mortality predictors in 498,103 UK Biobank participants: a prospective population-based study. *Lancet* **386**, 533–40 (2015). 10.1016/S0140-6736(15)60175-1 26049253

